# Bcl11a Controls Flt3 Expression in Early Hematopoietic Progenitors and Is Required for pDC Development *In Vivo*

**DOI:** 10.1371/journal.pone.0064800

**Published:** 2013-05-31

**Authors:** Xiaodi Wu, Ansuman T. Satpathy, Wumesh KC, Pentao Liu, Theresa L. Murphy, Kenneth M. Murphy

**Affiliations:** 1 Department of Pathology and Immunology, Washington University School of Medicine, St. Louis, Missouri, United States of America; 2 Wellcome Trust Sanger Institute, Hinxton, Cambridge, United Kingdom; 3 Howard Hughes Medical Institute, Washington University School of Medicine, St. Louis, Missouri, United States of America; Oklahoma Medical Research Foundation, United States of America

## Abstract

Bcl11a is a transcription factor known to regulate lymphoid and erythroid development. Recent bioinformatic analysis of global gene expression patterns has suggested a role for Bcl11a in the development of dendritic cell (DC) lineages. We tested this hypothesis by analyzing the development of DC and other lineages in *Bcl11a* ^−/−^ mice. We found that Bcl11a was required for expression of IL-7 receptor (IL-7R) and Flt3 in early hematopoietic progenitor cells. In addition, we found severely decreased numbers of plasmacytoid dendritic cells (pDCs) in *Bcl11a* ^−/−^ fetal livers and in the bone marrow of *Bcl11a* ^−/−^ fetal liver chimeras. Moreover, *Bcl11a* ^−/−^ cells showed severely impaired *in vitro* development of Flt3L-derived pDCs and classical DCs (cDCs). In contrast, we found normal *in vitro* development of DCs from *Bcl11a* ^−/−^ fetal liver cells treated with GM-CSF. These results suggest that the persistent cDC development observed in *Bcl11a* ^−/−^ fetal liver chimeras reflects derivation from a Bcl11a- and Flt3-independent pathway *in vivo*.

## Introduction

Dendritic cells (DCs), comprising classical DCs (cDCs) and plasmacytoid DCs (pDCs), develop from a common DC progenitor (CDP) residing in the bone marrow (BM); unlike myeloid progenitors at earlier stages of development, CDPs have excluded monocyte and macrophage potential but give rise to all DC subsets at the clonal level [1]–[4]. Several transcription factors that act broadly in hematopoiesis are known to regulate the development of all DCs, including Ikaros [5], [6], PU.1 [7], [8], and Gfi1 [9]. Transcription factors that regulate specific subsets of DCs have also been reported. For example, E2-2 is required for development of pDCs [10], Batf3 for CD8^+^ cDCs [11], Irf8 for pDCs and CD8^+^ cDCs [12], [13], and the NF-κB family member RelB for CD4^+^ cDCs [5], [14], [15].

A bioinformatic analysis of global gene expression patterns has identified groups of transcription factors that may be involved in fate decisions along the DC lineage [16]. Among genes that increase in expression from the macrophage–DC progenitor (MDP) to the CDP, those that do not increase in expression from the MDP to the monocyte were labeled in that analysis as possible promoters of DC commitment. Transcription factors identified by these criteria include some previously associated only with pDC development, including E2-2 and Spi-B [17], [18], and some previously associated only with cDCs, including Zbtb46 [19], [20]. Other factors identified in this analysis include Irf8, Bcl11a, and Runx2. Recently, it has been demonstrated in the setting of competitive BM reconstitution that Irf8 promotes the development of all DC subsets [21], even though *Irf8* ^−/−^ mice in other settings do not show defects in CD4^+^ cDC development [12], [13]. We wondered, therefore, whether a similar early role in DC development could be identified for another factor such as Bcl11a.

*Bcl11a* was first described as a gene located at a common proviral integration site in BXH2 myeloid leukemias, and its human ortholog was found to be a recurrent target of translocations in B cell malignancies [22], [23]. This gene encodes a Krüppel-like zinc finger transcription factor expressed in neural and lymphoid tissues that is essential for the development of B cells and for thymocyte maturation [24]. In the erythroid lineage, BCL11A acts *in trans* to silence the fetal hemoglobin locus in cooperation with the transcription factor SOX6 [25], [26]. Indeed, differences in stage-specific expression between human BCL11A and mouse Bcl11a account at least in part for interspecies differences in fetal hemoglobin expression patterns [25].

Although *Bcl11a* has been recognized as a useful marker of pDCs [27], [28], its actual role in DC development remains unreported. Thus, we sought to examine DC development in the setting of Bcl11a deficiency *in vivo* and *in vitro*. We found that Bcl11a was required for normal expression of IL-7 receptor (IL-7R) as well as Flt3 in early hematopoietic progenitors. In addition, we observed a strict requirement for Bcl11a in pDC development and found evidence for a Bcl11a-independent pathway of cDC development *in vivo*.

## Results

### Bcl11a is Required for Development of CLPs and CDPs

**Figure 1:**
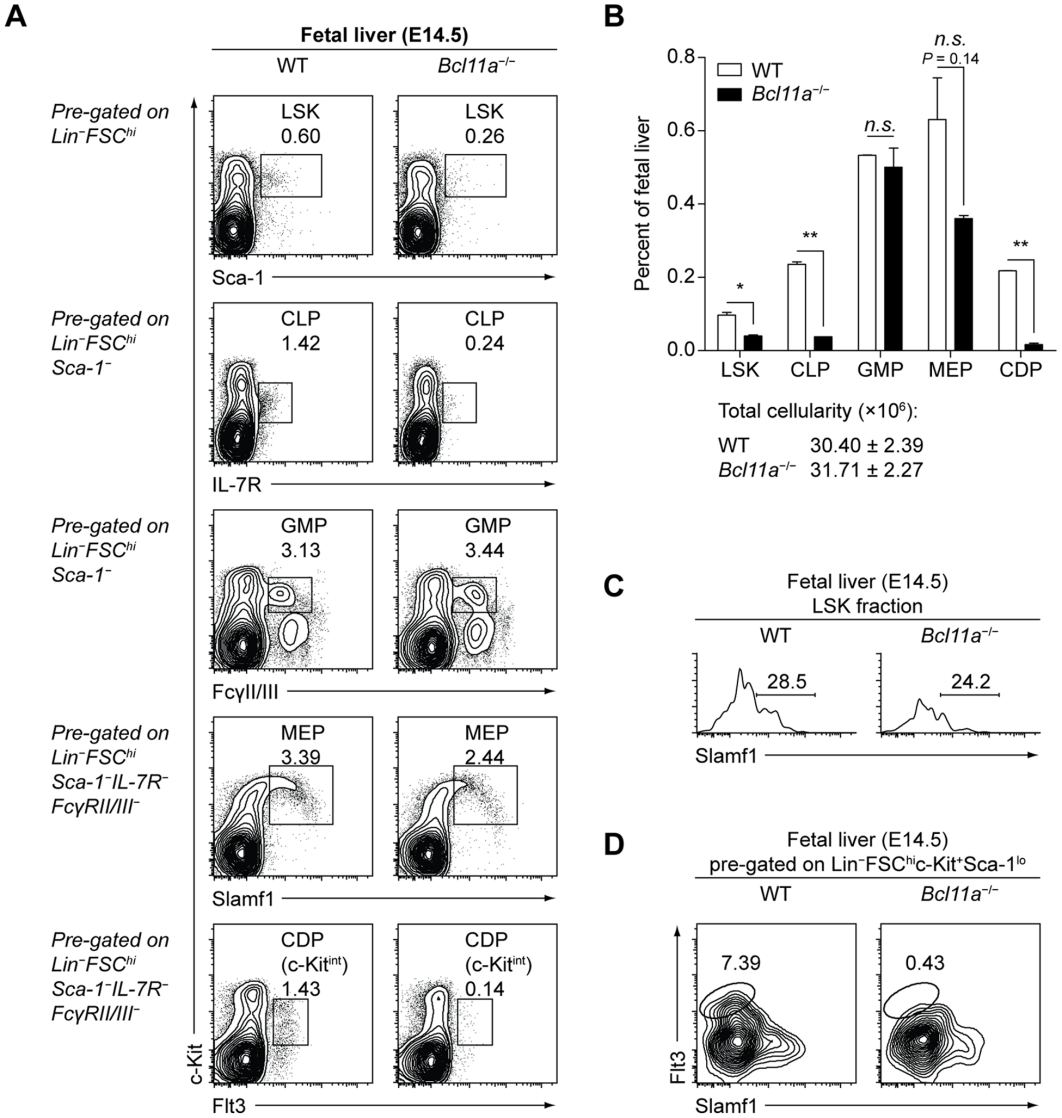
Bcl11a is required for development of lymphoid and DC progenitors in the fetus. (A) Flow cytometry analysis of progenitor populations in WT and *Bcl11a* ^−/−^ fetal livers dissected at embryonic day 14.5 (E14.5). Populations are gated as indicated; numbers represent the percentage of cells within the histogram that lie in the indicated gate. Data are representative of two mice per group. (B) Progenitor populations in WT and *Bcl11a* ^−/−^ fetal livers at E14.5, analyzed by flow cytometry as in (A) and presented as a percentage of total fetal liver cells. Bars represent the mean (± SEM) of two mice per group. (C) CD150 (Slamf1) expression within the LSK fraction in WT and *Bcl11a* ^−/−^ fetal livers at E14.5. (D) SL-GMPs in WT and *Bcl11a* ^−/−^ fetal livers at E14.5.

**Figure 2:**
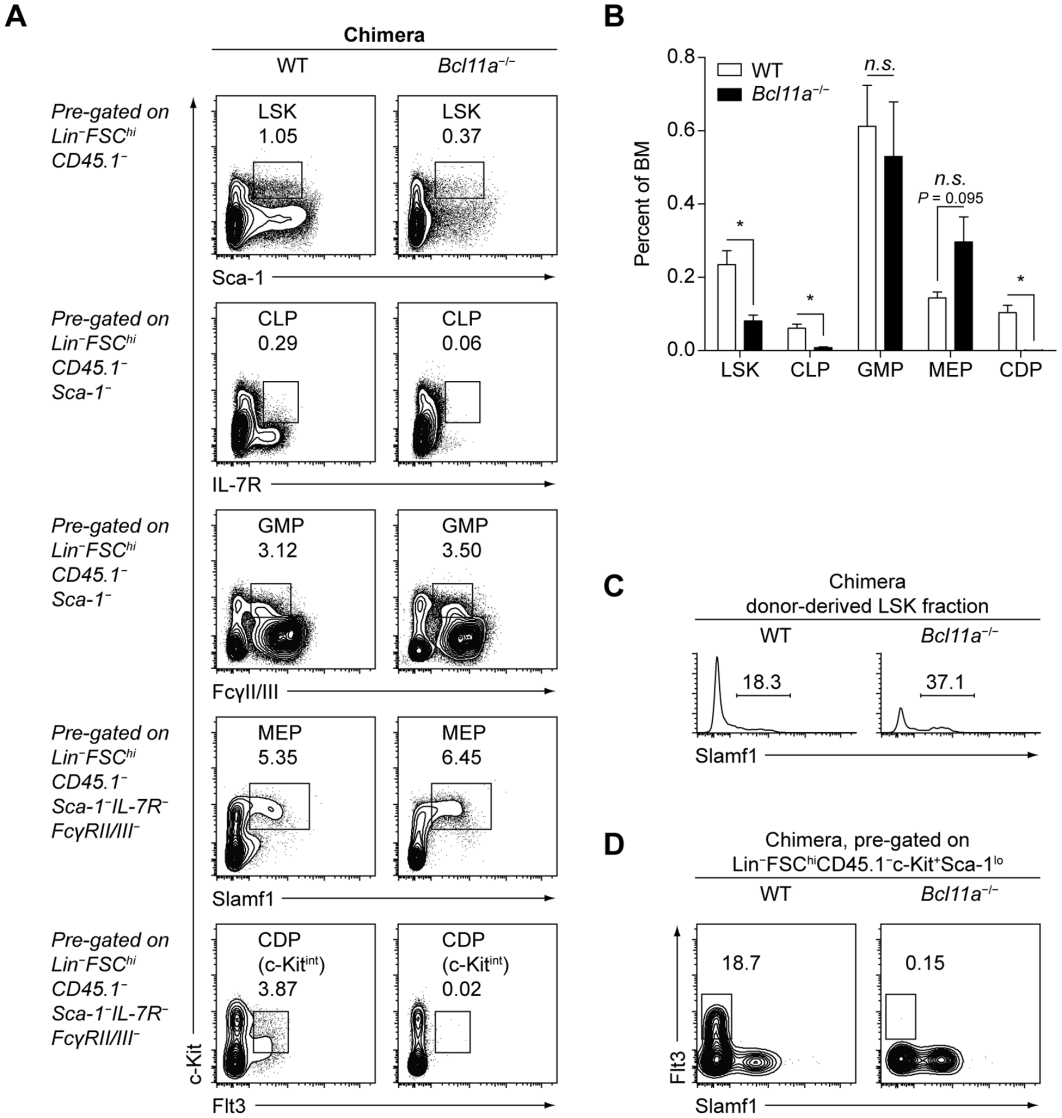
Bcl11a is required for development of lymphoid and DC progenitors in the adult. (A) Flow cytometry analysis of progenitor populations in lethally irradiated congenic mice reconstituted with WT or *Bcl11a* ^−/−^ fetal liver cells, analyzed four weeks after transplant. Data are representative of three mice per group. (B) Progenitor populations in WT and *Bcl11a* ^−/−^ fetal liver chimeras at four weeks after transplant, analyzed by flow cytometry as in (A) and presented as a percentage of total BM cells. Bars represent the mean (± SEM) of three mice per group. (C) CD150 (Slamf1) expression within the donor-derived LSK fraction in WT and *Bcl11a* ^−/−^ fetal liver chimeras at four weeks after transplant. (D) SL-GMPs in WT and *Bcl11a* ^−/−^ fetal liver chimeras at four weeks after transplant.

During hematopoiesis, *Bcl11a* is expressed at similar levels in the hematopoietic stem cell (HSC), multipotent progenitor (MPP), common lymphoid progenitor (CLP), common myeloid progenitor (CMP), and megakaryocyte–erythroid progenitor (MEP) [16]. To study the function of Bcl11a in hematopoietic progenitors, we used mice targeted for deletion of the first exon of *Bcl11a* [24]. Since *Bcl11a* ^−/−^ mice die *in utero* or perinatally, we compared hematopoietic progenitor populations present in wild type (WT) and *Bcl11a* ^−/−^ fetal livers at embryonic day 14.5. First, we analyzed development of Lin^−^Sca-1^+^c-Kit^+^ (LSK), CLP, granulocyte-macrophage progenitor (GMP), MEP, and CDP populations (**Fig. 1**). WT and *Bcl11a* ^−/−^ fetal livers showed comparable frequencies of GMPs and MEPs. However, *Bcl11a* ^−/−^ fetal livers showed a greater than twofold decrease in the frequency of LSK cells and more marked decreases in frequencies of IL-7R^+^ CLPs and Flt3^+^ CDPs relative to WT fetal livers (**Fig. 1A, B**); within the LSK fraction, *Bcl11a* ^−/−^ fetal livers showed defects in both CD150 (Slamf1)^+^ and CD150^–^ populations (**Fig. 1C**). One study has demonstrated that a Sca-1^lo^c-Kit^+^Flt3^+^CD150^–^ population with granulocyte and macrophage potential (SL-GMP) can be identified which excludes mast cell potential [29]; GMPs in the *Bcl11a* ^−/−^ fetal liver, however, lacked Flt3 expression (data not shown) and no SL-GMP population could be identified (**Fig. 1D**). Next, we analyzed hematopoietic development in chimeras produced by transferring WT or *Bcl11a* ^−/−^ fetal liver cells into lethally irradiated congenic recipient mice (**Fig. 2**). Four to six weeks after transfer, donor-derived *Bcl11a* ^−/−^ BM showed decreased frequencies of LSK cells, CLPs, and CDPs but comparable frequencies of GMPs and MEPs relative to donor-derived WT BM (**Fig. 2A, B**); within the LSK fraction, donor-derived *Bcl11a* ^−/−^ BM showed a greater proportion of CD150^+^ cells than did donor-derived WT BM, corresponding to a decrease in the overall frequency of the more differentiated CD150^–^ population (**Fig. 2C**). As in *Bcl11a* ^−/−^ fetal livers, no SL-GMP population could be identified in donor-derived *Bcl11a* ^−/−^ BM (**Fig. 2D**). In summary, the loss of Bcl11a in hematopoietic progenitors resulted in impaired development of LSK cells as well as a selective loss of CLPs and CDPs; these effects were observed both in the fetal stage and in the adult chimera, demonstrating that this factor is required in fetal and adult hematopoiesis.

Conceivably, the absence of IL-7R^+^ CLPs and Flt3^+^ CDPs in *Bcl11a* ^−/−^ fetal livers and BM could result from a requirement for Bcl11a in the development of the CLP and CDP or from a more restricted requirement for Bcl11a in the expression of IL-7R and Flt3, the surface markers that identify these populations. In either case, however, the loss of Bcl11a should result in DC defects because Flt3 ligand (Flt3L) signaling is essential for DC development in the steady state [30]–[32].

### Bcl11a Regulates Expression of *Il7r* and *Flt3*

**Figure 3:**
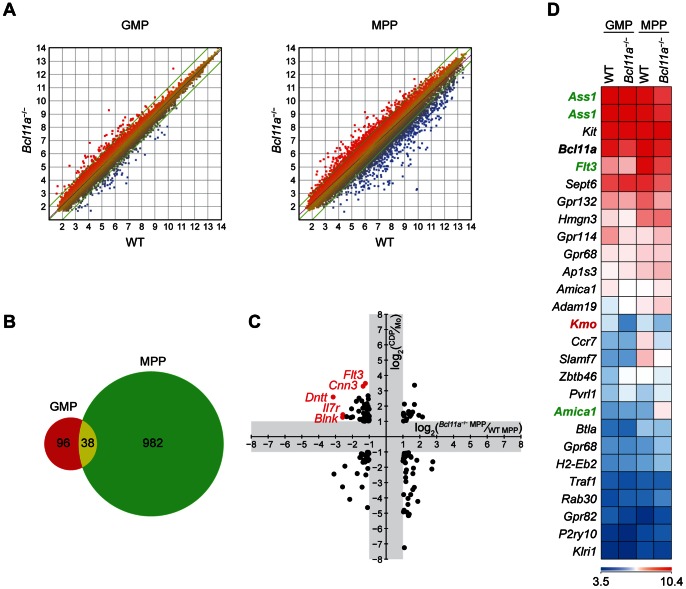
Bcl11a regulates the expression of *Flt3* and *Il7r*. (A) Microarray analysis of sorted GMPs (left) and MPPs (right) from WT and *Bcl11a* ^−/−^ fetal liver chimeras. (B) Shown is a Venn diagram of probe sets (excluding normalization controls) with a greater than twofold change in expression between WT and *Bcl11a* ^−/−^ MPPs. (C) Shown are log_2_-transformed ratios of gene expression in *Bcl11a* ^−/−^ MPPs relative to WT MPPs (*x*-axis) plotted against log_2_-transformed ratios of gene expression in WT CDPs relative to WT monocytes (ImmGen; *y*-axis). For clarity, probe sets with less than twofold changes in expression (log_2_-transformed ratios between −1 and 1) along either dimension are omitted (gray). (D) Shown is a heat map of log_2_-transformed gene expression in WT and *Bcl11a* ^−/−^ GMPs and MPPs for probe sets that constitute an ImmGen core cDC signature. Highlighted are genes that show a greater than twofold change in expression between WT and *Bcl11a* ^−/−^ GMPs (red) or between WT and *Bcl11a* ^−/−^ MPPs (green).

To identify Bcl11a target genes that explain its role in hematopoietic progenitors, we compared global gene expression by microarray for donor-derived WT and *Bcl11a* ^−/−^ populations isolated from chimeric BM (**Fig. 3**). Since we observed that IL-7R- and Flt3-expressing populations were affected by the loss of Bcl11a, we avoided the use of these surface markers in order to allow for comparison of equivalent populations across genotypes. Thus, we isolated multipotent progenitors (MPPs) as identified by the lack of CD150 expression within the LSK fraction [33]–[35]. We also isolated GMPs from the same BM, since the size of this population was unaffected by loss of Bcl11a.

We found that WT and *Bcl11a* ^−/−^ GMPs were more similar to each other in gene expression than WT and *Bcl11a* ^−/−^ MPPs were to each other (**Fig. 3A**). One hundred and thirty-four probe sets showed a greater than twofold change in expression between WT and *Bcl11a* ^−/−^ GMPs. In contrast, 1020 probe sets showed a greater than twofold change in expression between WT and *Bcl11a* ^−/−^ MPPs; of these, only 38 also show a greater than twofold change between WT and *Bcl11a* ^−/−^ GMPs (**Fig. 3B**). These data suggest that GMP population size is unaffected by loss of Bcl11a because this transcription factor regulates relatively few genes in GMPs.

Since the loss of Bcl11a impaired development of CDPs but not GMPs, we examined Bcl11a target genes which showed expression patterns that distinguish DCs from monocytes and macrophages. Thus, we compared the ratio of gene expression in CDPs relative to monocytes against the ratio of gene expression in *Bcl11a* ^−/−^ MPPs relative to WT MPPs (**Fig. 3C**). Of genes most highly expressed in CDPs relative to monocytes, those most affected by loss of Bcl11a included *Flt3*, *Cnn3* (encoding calponin 3), *Dntt* (encoding the template-independent DNA polymerase TdT), *Il7r*, and *Blnk* (encoding B-cell linker protein, which links components of B-cell receptor signaling). We also compared changes in gene expression between WT and *Bcl11a* ^−/−^ MPPs for members of the core cDC transcriptional signature identified in a published bioinformatic analysis [16] (**Fig. 3D**). Within this core signature, we found only three genes–*Ass1*, *Amica1*, and *Flt3*–that showed a greater than twofold decrease in expression in *Bcl11a* ^−/−^ MPPs relative to WT MPPs. Taken together, the decreased expression of *Flt3* and *Il7r* in *Bcl11a* ^−/−^ MPPs suggests that Bcl11a may be specifically required for the expression of these genes.

### Bcl11a is Required for pDC but not cDC Development *in vivo*

**Figure 4:**
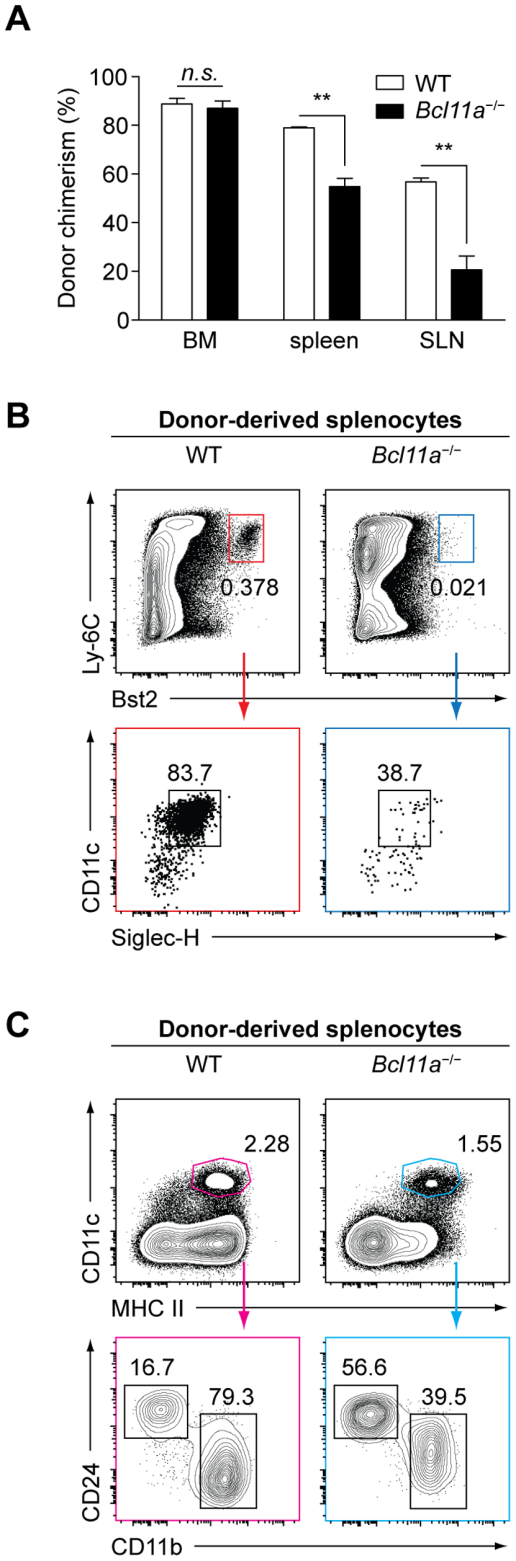
Bcl11a is required *in vivo* for development of pDCs but not cDCs. (A) Donor-derived (CD45.2^+^) chimerism in the BM, spleen, and skin-draining lymph node (SLN) of WT and *Bcl11a* ^−/−^ fetal liver chimeras. Bars represent the mean (± SEM) of three mice per group. (B) Flow cytometry analysis of donor-derived pDCs in the spleen. Data are representative of three mice per group. (C) Flow cytometry analysis of donor-derived cDCs in the spleen. Data are representative of three mice per group.

Next, we examined the development of mature hematopoietic subsets in WT and *Bcl11a* ^−/−^ fetal liver chimeras (**Fig. 4**). In accordance with previous reports [24], we observed atrophic thymi in *Bcl11a* ^−/−^ chimeras (data not shown). In the BM, the size of the donor-derived compartment was comparable in WT and *Bcl11a* ^−/−^ chimeras; in the spleen and skin-draining lymph nodes, *Bcl11a* ^−/−^ cells were somewhat impaired in their competition against the residual host population (**Fig. 4A**). Within the donor-derived compartment of the spleen, a profound defect in pDC development was apparent in *Bcl11a* ^−/−^ chimeras relative to WT chimeras (**Fig. 4B**). In contrast, donor-derived cDCs were present in *Bcl11a* ^−/−^ chimeras with no significant decrease relative to WT chimeras (**Fig. 4C**).

**Figure 5:**
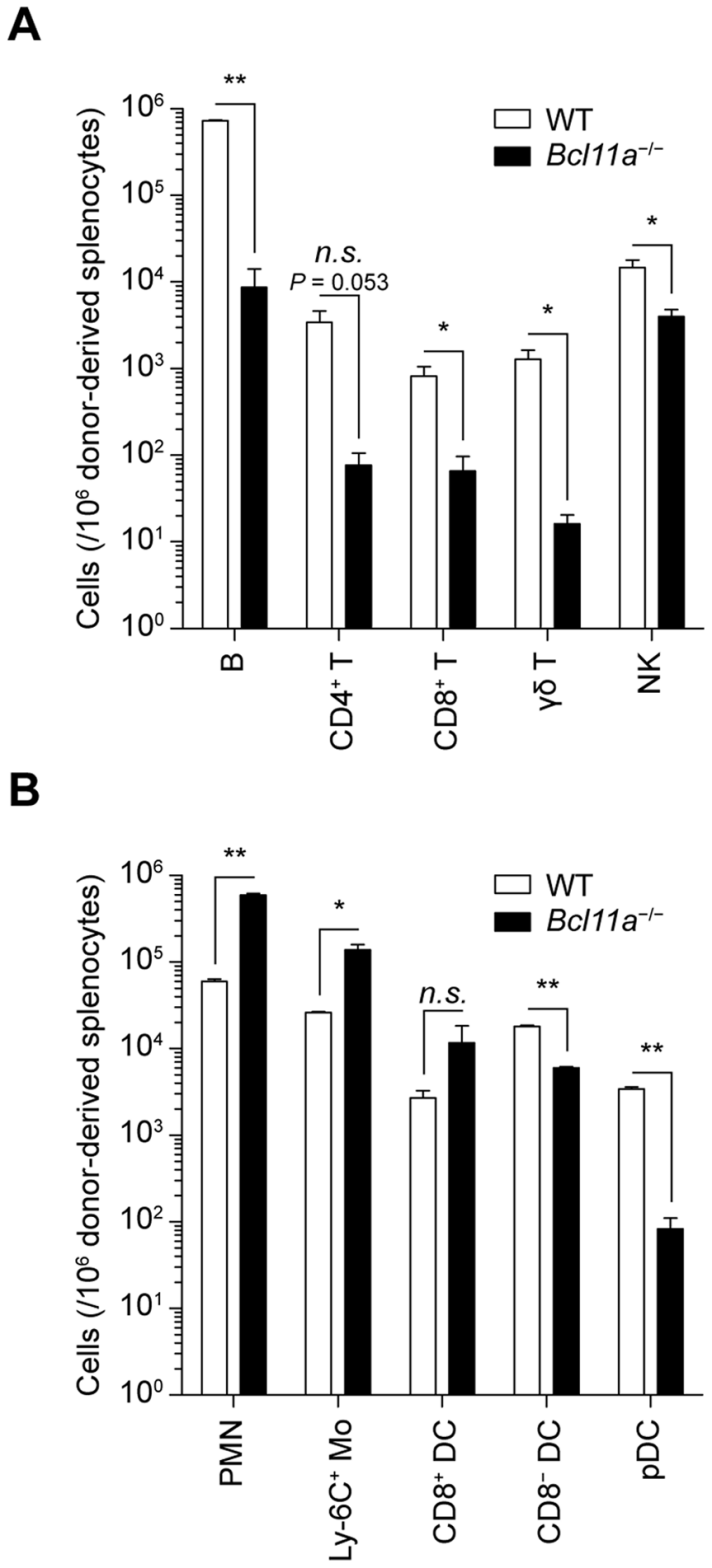
Bcl11a deficiency *in vivo* impairs development of lymphoid and myeloid populations. (A) Donor-derived lymphoid populations in the spleen of WT and *Bcl11a* ^−/−^ fetal liver chimeras, analyzed by flow cytometry. Bars represent the mean (± SEM) of three mice per group. (B) Donor-derived myeloid populations in the spleen of WT and *Bcl11a* ^−/−^ fetal liver chimeras, analyzed by flow cytometry as in Fig. 4. Bars represent the mean (± SEM) of three mice per group.

Among lymphoid subsets, donor-derived B cells, CD4 T cells, CD8 T cells, and γδ T cells were decreased in frequency by at least tenfold in the spleens of *Bcl11a* ^−/−^ chimeras as compared to WT chimeras, consistent with previous reports [24], while NK cells were decreased by slightly more than threefold (**Fig. 5A**). Among myeloid subsets other than pDCs, donor-derived CD8^−^ cDCs showed a modest threefold decrease in the spleens of *Bcl11a* ^−/−^ chimeras as compared to WT chimeras; other myeloid populations examined, including CD8^+^ cDCs, were not decreased in frequency (**Fig. 5B**). Thus, Bcl11a was strictly required for the development of pDCs but not cDCs *in vivo*.

### Flt3-dependent, but not GM-CSF–dependent, DC Development Requires Bcl11a *in vitro*

**Figure 6:**
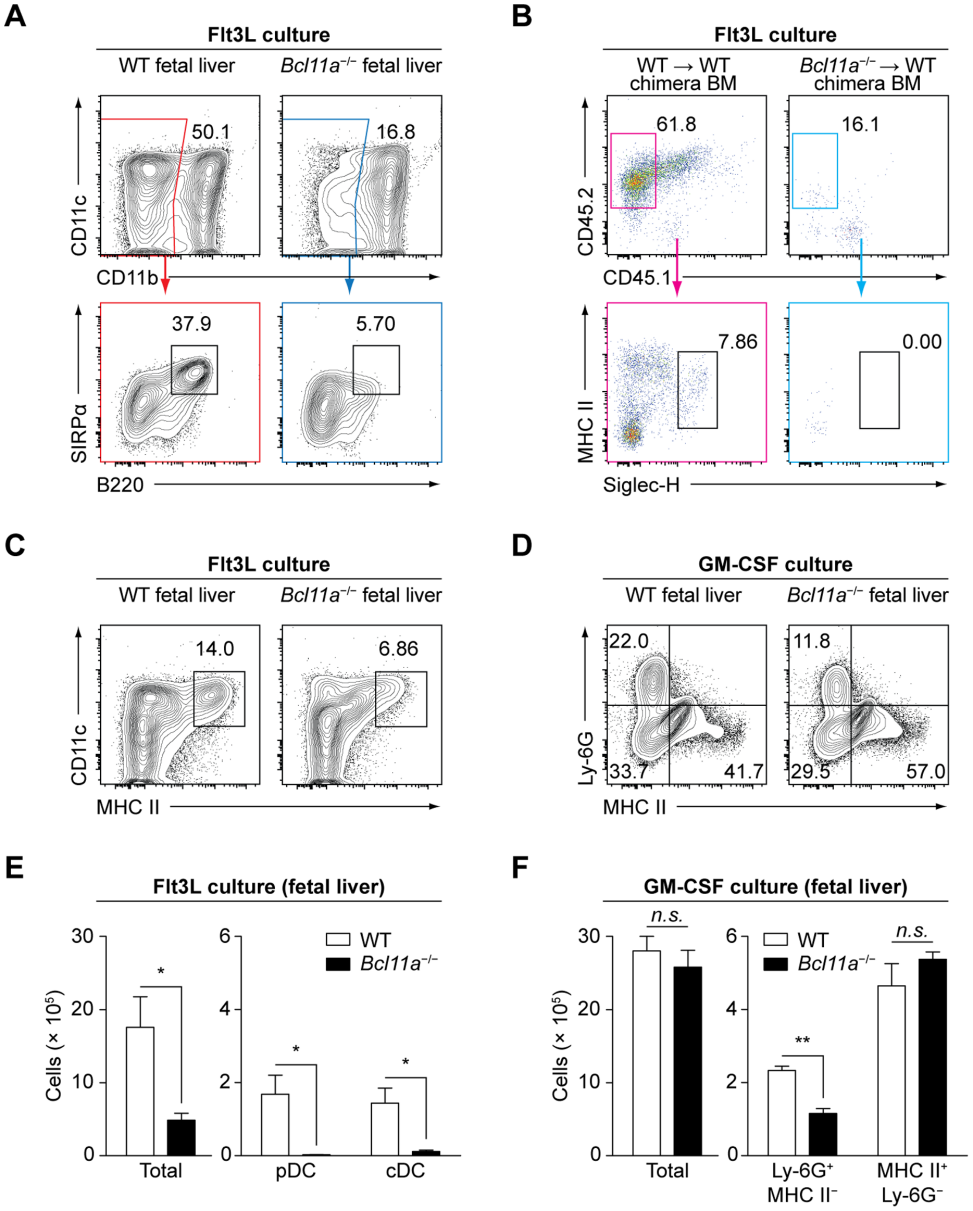
Bcl11a is required *in vitro* for development of Flt3L-derived pDCs and cDCs but not GM-CSF–derived cDCs. (A) Flow cytometry analysis of pDCs in Flt3L cultures of fetal liver cells. Data are representative of three to four replicates over two experiments. (B) Flow cytometry analysis of pDCs in Flt3L cultures of BM cells derived from fetal liver chimeras. Data are representative of three replicates. (C, D) Flow cytometry analysis of Flt3L-derived cDCs (C) or GM-CSF-derived DCs (D) in cultures of fetal liver cells. Data are representative of three to four replicates over two experiments. (E, F) Counts of total cells and indicated subsets in Flt3L cultures (E) or GM-CSF cultures (F) of fetal liver cells, analyzed by flow cytometry as in (C) or (D), respectively. Bars represent the mean (± SEM) of three to four replicates per group pooled from two experiments.

We compared the development of WT and *Bcl11a* ^−/−^ cells *in vitro* in response to treatment with Flt3L or granulocyte macrophage colony-stimulating factor (GM-CSF) (**Fig. 6**). The observation that *Flt3* ^−/−^ mice retain DC development [36] suggests an alternative receptor for Flt3L or a Flt3L-independent pathway for DC development. Thus, we supplied excess Flt3L or GM-CSF to distinguish between these possibilities in the context of Bcl11a deficiency. As expected, pDCs developed from WT fetal liver cells (**Fig. 6A**) and from the donor-derived BM cells of WT chimeras (**Fig. 6B**) in response to Flt3L treatment. In contrast, pDCs developed in markedly decreased numbers from *Bcl11a* ^−/−^ fetal liver cells and completely failed to develop from the donor-derived BM cells of *Bcl11a* ^−/−^ chimeras under these conditions (**Fig. 6A, B**), demonstrating that Bcl11a is required for pDC development in response to Flt3L both *in vivo* and *in vitro*. We also examined cDC development from WT and *Bcl11a* ^−/−^ fetal liver cells *in vitro* in response to treatment with Flt3L or GM-CSF. Flt3L-derived cDCs were markedly reduced in cultures of *Bcl11a* ^−/−^ fetal liver cells relative to cultures of WT fetal liver cells (**Fig. 6C, E**). However, GM-CSF–derived DCs developed in normal numbers from cultures of *Bcl11a* ^−/−^ fetal liver cells relative to cultures of WT fetal liver cells (**Fig. 6D, F**). These results suggest that Flt3L cannot signal through an alternative receptor to rescue cDC development in *Bcl11a* ^−/−^ cells, but that an alternative pathway of DC development may be mediated by GM-CSF.

### Loss of Flt3L Results in Lineage-specific Defects in pDC Development

**Figure 7:**
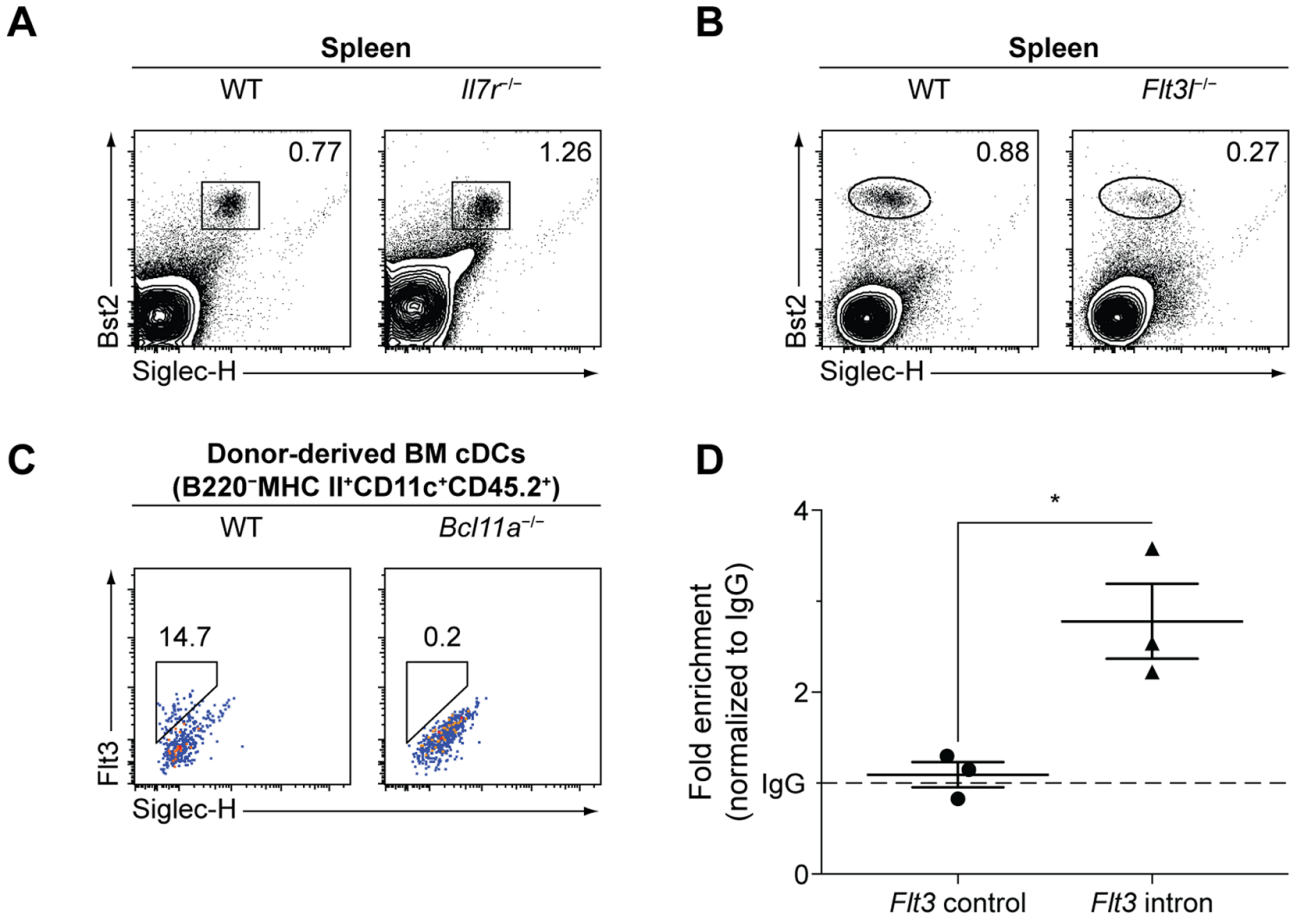
Cytokine signaling in DC development and regulation by Bcl11a. (A) Flow cytometry analysis of pDCs in WT and *Il7r* ^−/−^ spleens. Data are representative of four mice per group over two experiments. (B) Flow cytometry analysis of pDCs in WT and *Flt3l* ^−/−^ spleens. Data are representative of three mice per group over two experiments. (C) Flow cytometry analysis of donor-derived cDCs in the spleen of WT and *Bcl11a* ^−/−^ fetal liver chimeras, analyzed by flow cytometry as in Fig. 4. Data are representative of three mice per group. (D) Bcl11a binding in the *Flt3* genomic locus assayed by ChIP-qPCR. Data are represented as fold enrichment as compared to isotype control.

Next, we examined the development of splenic pDCs in the context of IL-7R or Flt3L deficiency. A previous study has demonstrated that splenic pDCs in *Il7r* ^−/−^ mice or *Il7* ^−/−^ mice are decreased in absolute number when compared to WT controls [37]. We found that splenic pDCs in *Il7r* ^−/−^ mice were not decreased in proportion to total splenocytes when compared to WT controls matched for strain, age, and sex (**Fig. 7A**). This result suggests that the hematopoietic defects in these mice may have relatively few lineage-specific consequences for pDC development.

Previously, it has been found that *Flt3* ^−/−^ mice and *Flt3l* ^−/−^ mice show defects in the development of pDCs [36], [38]. Accordingly, and in contrast to our observations in *Il7r* ^−/−^ mice, we found that *Flt3l* ^−/−^ mice showed a greater than fourfold reduction in splenic pDC frequency as compared to WT littermate controls (**Fig. 7B**), in addition to reductions in absolute spleen size (data not shown).

### Bcl11a is Required for Flt3 Expression in cDCs and Binds the *Flt3* Genomic Locus

Because we observed cDC development in *Bcl11a* ^−/−^ chimeras, we assessed whether these cells might express Flt3 in a Bcl11a-independent manner; however, unlike WT cDCs in the BM, *Bcl11a* ^−/−^ cDCs in the same compartment showed no discernible Flt3 expression by flow cytometry (**Fig. 7C**), again suggesting that a Flt3-independent pathway is instead responsible for their development. Finally, to assay Bcl11a binding at the *Flt3* locus, we performed chromatin immunoprecipitation (ChIP) using mouse pro-B cells. By quantitative real-time polymerase chain reaction (qPCR), we detected an approximately threefold enrichment at a region in the first intron of the *Flt3* locus in DNA precipitated using anti-Bcl11a antibody as compared to isotype control (**Fig. 7D**).

In summary, our results document a strict requirement for Bcl11a in pDC development both *in vivo* and *in vitro*; further, the requirement for Bcl11a in cDC development may differ based on the cytokine stimulus to which progenitors are exposed. The actions of Bcl11a include regulation of Flt3 expression by direct binding to the *Flt3* locus, and Bcl11a is required for Flt3 expression in DCs and their progenitors.

## Discussion

This study extends the known actions of *Bcl1la* in immune lineage development and provides a mechanism for its effects. Although *Bcl11a* has been recognized as a factor required for normal lymphoid development [24], the basis for this requirement has been unclear. It has been shown that Bcl11a acts upstream of the B cell factors Ebf1 and Pax5 and that *Il7r* mRNA is not expressed in *Bcl11a* ^−/−^ fetal livers [24]. Lack of IL-7Rα or the cytokine receptor common γ chain (γ_c_, encoded by *Il2rg*) severely impairs T and B cell development [39]–[43]. In T cell development, IL-7R signaling is thought to promote thymocyte survival, since Bcl-2 rescues impaired T cell development in *Il7r* ^−/−^ or *Il2rg* ^−/−^ mice [44]–[48]. In B cell development, Bcl-2 does not rescue development in the absence of IL-7R or γ_c_ [44]–[47], [49], and IL-7R signaling is thought to induce expression of the transcription factor Ebf in the CLP [50]–[52].

Here, we demonstrate that Bcl11a is required for normal expression of IL-7R as early as the CLP and we add the novel observation that Bcl11a promotes the development of Flt3-dependent lineages. Together, these actions provide a more complete account for previously observed defects in lymphocyte development in *Bcl11a* ^−/−^ mice, since T cell potential is preserved in IL-7–deficient CLPs in a Flt3L-dependent manner [53], [54]. The mechanisms by which Bcl11a deficiency impairs T and B cell development, however, still remain incompletely explored. Consistent with a previous report [24], we confirmed the presence of residual T and B cells in the spleen of chimeras reconstituted with *Bcl11a* ^−/−^ fetal liver cells. By contrast, tamoxifen-induced deletion of *Bcl11a* in chimeras that have been reconstituted with *Rosa26-CreERT2*;*Bcl11a^flox^* ^/*flox*^ BM cells results in a more profound loss of T and B cells [55]. Thus, synchronous deletion of *Bcl11a* within a previously intact hematopoietic compartment produces a different outcome than does sustained deficiency throughout hematopoiesis. These results may point to a crucial lymphopoietic role for cells in which Bcl11a is dispensable for survival but necessary for development or maturation, or vice versa; these cells could include HSCs, mature T and B cells, or even residual CLPs undetectable due to a lack of IL-7R and Flt3 expression.

In line with a previous finding that E2-2 regulates *Bcl11a* expression [18], we also document a strict requirement for Bcl11a in the development of pDCs. The development of pDCs *in vivo* was lost in *Bcl11a* ^−/−^ fetal liver chimeras. In agreement, Flt3L cultures of BM derived from these *Bcl11a* ^−/−^ chimeras showed a complete loss of pDC development *in vitro*. Because mature pDCs are short-lived, non-proliferative, and continuously replenished from progenitor populations [56], [57], the nearly complete loss of this population is most attributable to a developmental defect and not merely to cell survival defects in mature pDCs. This interpretation would be consistent with a finding that rescue of Bcl11a-deficient progenitors from increased apoptosis by p53 deficiency is unable to restore lymphoid potential [55].

Notably, however, *in vitro* development of cDCs was eliminated in Flt3L cultures of *Bcl11a* ^−/−^ fetal liver cells but was maintained in GM-CSF cultures of *Bcl11a* ^−/−^ fetal liver cells. Flt3L and GM-CSF have distinct, non-redundant actions in supporting cDC development [58], [59]. The combined loss of Flt3L and GM-CSF causes a more severe cDC deficiency than loss of Flt3L alone; indeed, Flt3L-deficient mice retain an appreciable population of Flt3-expressing progenitors [38]. The maintenance of cDCs in *Bcl11a* ^−/−^ fetal liver chimeras suggests that these cells may rely on a Bcl11a- and Flt3-independent pathway for their development, survival, or expansion. Conditional knockout models would clarify which of these alternatives underlie the observed phenotype in *Bcl11a* ^−/−^ mice. Since DCs developed normally *in vitro* from *Bcl11a* ^−/−^ progenitors treated with GM-CSF, it is possible that *Bcl11a* ^−/−^ cDCs *in vivo* indeed represent development from GM-CSF–dependent progenitors, related perhaps to monocyte-derived dendritic cell lineages [60].

## Materials and Methods

### Ethics Statement

This study was carried out in strict accordance with recommendations in the U.S. National Research Council *Guide for the Care and Use of Laboratory Animals*. The protocol was approved by the Washington University Animal Studies Committee (#20090320).

### Mice

C57BL/6, B6.SJL, *Il7* ^−/−^, and *Il7r* ^−/−^ mice were purchased from The Jackson Laboratory. *Flt3l* ^−/−^ and *Rag2* ^−/−^ mice were purchased from Taconic Farms. *Flt3l* ^−/−^ mice were subsequently crossed to *Zbtb46^gfp^* ^/*gfp*^ mice generated previously [19]; F2 offspring were studied in the present experiments, with *Zbtb46* ^+/*gfp*^;*Flt3l* ^+/+^ or *Zbtb46^gfp^* ^/*gfp*^;*Flt3l* ^+/+^ mice used as WT littermate controls. *Bcl11a* ^−/−^ mice were obtained from Dr. Pentao Liu [24]. Mice were bred and maintained in our specific pathogen free animal facility at Washington University in St. Louis. Mice were sacrificed by CO_2_ overdose followed by cervical dislocation.

### Single-cell Suspensions of Fetal Liver

At embryonic day 14.5, fetal livers were mechanically dissociated with a syringe plunger and sterile 70-µm cell strainer (Fisher) into IMDM +10% (v/v) FCS (I10F). For subsequent cell culture or flow cytometry, red blood cells were lysed in ACK lysing buffer before counting by Vi-CELL (Beckman Coulter).

### Antibodies

The following antibodies were purchased from BD Biosciences: FITC anti-CD3e (145-2C11), APC anti-CD4 (RM4-5), V450 anti-CD4 (RM4-5), PerCP-Cy5.5 anti-CD8a (53-6.7), PerCP-Cy5.5 anti-CD11b (M1/70), APC anti-CD11c (HL3), APC anti-CD19 (1D3), PE-Cy7 anti-CD24 (M1/69), APC anti-CD25 (PC61), FITC anti-CD45 (30-F11), APC anti-CD45.2 (104), APC-Cy7 anti-CD45.2 (104), PE anti-CD135 (A2F10.1), APC anti-CD172a (P84), FITC anti-B220 (RA3-6B2), V500 anti-B220 (RA3-6B2), PE anti-Gr-1 (RB6-8C5), V450 anti-Gr-1 (RB6-8C5), PerCP-Cy5.5 anti-IgM (R6-60.2), PE-Cy7 anti-Ly-6A/E (Sca-1) (D7), FITC anti-Ly-6C (AL-21), V450 anti-Ly-6C (AL-21), PE anti-Ly-6G (1A8), PE anti-MHC II (I-A/I-E) (M5/114.15.2), PE anti-TCRγδ (GL3). The following antibodies were purchased from eBioscience: APC-eFluor 780 anti-CD11c (N418), eFluor 450 anti-CD11c (N418), PerCP-Cy5.5 anti-CD16/32 (93), APC-eFluor 780 anti-CD44 (IM7), biotin anti-CD45.1 (A20), PerCP-Cy5.5 anti-CD45.1 (A20), Alexa Fluor 700 anti-CD45.2 (104), PE-Cy7 anti-CD49b (DX5), PE anti-CD103 (2E7), APC-eFluor 780 anti-CD117 (ACK2), PE-Cy7 anti-CD117 (2B8), FITC anti-CD127 (A7R34), APC anti-CD150 (mShad150), eFluor 450 anti-B220 (RA3-6B2), PE-Cy7 anti-B220 (RA3-6B2), APC anti-BST2 (eBio927), eFluor 450 anti-BST2 (eBio927), FITC anti-F4/80 (BM8), PE anti-IgD (41239), eFluor 450 anti-MHC II (I-A/I-E) (M5/114.15.2), eFluor 450 anti-NKp46 (29A1.4), FITC anti-Siglec-H (eBio440C). The following antibodies were purchased from Caltag: FITC anti-CD8a (5H10), PE anti-B220 (RA3-6B2). Qdot 605 streptavidin was purchased from Invitrogen and V500 streptavidin was purchased from BD Biosciences.

### Flow Cytometry and Sorting

Staining was performed at 4°C in the presence of Fc block (clone 2.4G2, BD Biosciences or BioXCell) in FACS buffer (DPBS +0.5% BSA +2 mM EDTA). Cells were analyzed using a FACSCanto II (BD Biosciences) or sorted using a FACSAria (BD Biosciences); data were visualized using FlowJo software (TreeStar).

### Cell Cultures

Cells were diluted to 2×10^6^ cells/ml in I10F +20 ng/ml Flt3L or GM-CSF, cultured in 12-well plates for 10 d (Flt3L) or 7 d (GM-CSF), then analyzed by flow cytometry.

### Chimeras

B6.SJL mice were lethally irradiated (1200 rad) and injected intraorbitally with 4×10^6^ fetal liver cells isolated from WT or *Bcl11a* ^−/−^ fetuses. After 4 or 6 weeks, BM was isolated by grinding and Histopaque-1119 (Sigma-Aldrich) centrifugation and either sorted by flow cytometry or cultured. From these mice, thymi were minced and digested in 250 µg/ml collagenase B (Roche) and 30 U/ml DNase I (Sigma-Aldrich) and analyzed by flow cytometry.

### Microarray Analysis

MPP and GMP populations were sorted from fetal liver chimeras and pooled by donor genotype. RNA was isolated using an RNAqueous-Micro Kit (Ambion) and submitted for amplification, labeling and hybridization. Expression values were analyzed after RMA quantile normalization using ArrayStar software (DNASTAR). Data were deposited in the Gene Expression Omnibus (GEO) repository under accession no. GSE46270.

### ChIP-qPCR

Pro-B cell cultures were established using *Rag2* ^−/−^ BM isolated by flushing and resuspended in I10F +5 ng/ml IL-7. Chromatin was prepared from 1×10^7^ cultured pro-B cells sonicated using a Bioruptor (Diagenode), immunoprecipitation was performed with a rabbit polyclonal anti-Bcl11a antibody (NB600-261, Novus Biologicals) or control rabbit IgG, and qPCR analysis was carried out using SYBR Green-based detection and the following previously published primers [61]: *Flt3* control forward, 5′-TTTGCACTCGTAGCAAATGG-3′; *Flt3* control reverse, 5′-GTTCAGCTGCCAAAGAGAGG-3′; *Flt3* promoter forward, 5′-GTTCAGCTGCCAAAGAGAGG-3′; *Flt3* promoter reverse, 5′-CGTCACTGACCACAGATTCC-3′; *Flt3* intron forward, 5′-AAAAGAGGAACTATTGGTATTTCG-3′; *Flt3* intron reverse, 5′-TGACAGTAGTGAAAACACACACACA-3′.

### Statistics

Statistical differences were identified using Prism 6 (GraphPad) by multiple unpaired Student’s *t* tests, controlling the false discovery rate (Q) by the method of Benjamini and Hochberg. *, Q = 0.05; **, Q = 0.01.
